# Acetate Does Not Affect Palmitate Oxidation and AMPK Phosphorylation in Human Primary Skeletal Muscle Cells

**DOI:** 10.3389/fendo.2021.659928

**Published:** 2021-06-17

**Authors:** Manuel A. González Hernández, Ellen E. Blaak, Nicole T. H. Hoebers, Yvonne P. G. Essers, Emanuel E. Canfora, Johan W. E. Jocken

**Affiliations:** Department of Human Biology , School of Nutrition and Translational Research in Metabolism (NUTRIM), Maastricht University, Maastricht, Netherlands

**Keywords:** gut metabolite, acetate, fat oxidation, insulin sensitivity (IS), metabolic health

## Abstract

Our recent *in vivo* human studies showed that colonic administration of sodium acetate (SA) resulted in increased circulating acetate levels, which was accompanied by increments in whole-body fat oxidation in overweight-obese men. Since skeletal muscle has a major role in whole-body fat oxidation, we aimed to investigate effects of SA on fat oxidation and underlying mechanisms in human primary skeletal muscle cells (HSkMC). We investigated the dose (0–5 mmol/L) and time (1, 4, 20, and 24 h) effect of SA on complete and incomplete endogenous and exogenous oxidation of ^14^C-labeled palmitate in HSkMC derived from a lean insulin sensitive male donor. Both physiological (0.1 and 0.25 mmol/L) and supraphysiological (0.5, 1 and 5 mmol/L) concentrations of SA neither increased endogenous nor exogenous fat oxidation over time in HSkMC. In addition, no effect of SA was observed on *Thr^172^-AMPKα* phosphorylation. In conclusion, our previously observed *in vivo* effects of SA on whole-body fat oxidation in men may not be explained *via* direct effects on HSkMC fat oxidation. Nevertheless, SA-mediated effects on whole-body fat oxidation may be triggered by other mechanisms including gut-derived hormones or may occur in other metabolically active tissues.

## Introduction

In the last decades obesity has reached pandemic proportions worldwide (1), indicating the necessity to take action and prevent the development of obesity-related comorbidities. Obesity is characterized by an imbalance in energy intake and expenditure, with the excess energy stored in adipose tissue depots. It is increasingly clear that not adipose mass *per se* but rather adipose tissue dysfunction plays a central role in the observed metabolic derangements (1). A limited buffering capacity may result in systemic lipid overflow and an increased lipid supply to non-adipose tissues. Consequently, this may, together with an impaired oxidative capacity cause ectopic fat deposition in important metabolically active tissues (i.e. skeletal muscle, liver), which may link to the development of insulin resistance (2).

Of note, evidence is accumulating that energy and substrate metabolism and insulin sensitivity may be modulated by gut microbially derived metabolites, thus bringing attention to the cross-talk between gut-microbiota-derived metabolites and host metabolic health (3, 4). In the colon, fermentation of indigestible food compounds by the resident microbiota plays a pivotal role in the production of short-chain fatty acids (SCFA), including acetate, propionate, and butyrate (3, 5, 6). Of these SCFA, acetate is the most abundant in the colon and has potential beneficial effects on energy expenditure and substrate metabolism (7). Circulating levels of acetate can reach physiological concentrations that range between 5 up to an average of 450 µmol/L, predominantly after prebiotic consumption (4, 8–15). Subsequently, gut-derived acetate seems to have a broad metabolic role, since the SCFA-sensing G protein-coupled receptors (GPR, 41/43) are expressed in various tissues such as the adipose tissue (16), skeletal muscle, liver (17), and pancreatic beta cells (18, 19). Circulating acetate has been shown to have antilipolytic effects in adipose tissue in *in vitro* (20–22), animal (20, 23), and human studies (24–27), thus potentially reducing lipid overflow, improving lipid buffering capacity and impacting peripheral insulin sensitivity. Other potential metabolic effects may include the induction of satiety mediated *via* the stimulation of glucagon-like peptide 1 (GLP-1) and peptide YY (PYY) secretion (28, 29).

In addition to satiety, SCFA effects on intestinal gluconeogenesis and increased skeletal muscle fat oxidation have been observed at least in rodents (3). Furthermore, an *in vitro* study in L6 myotubes showed that acetate acutely (following 2 min) increased AMP-activated protein kinase (*AMPK)* phosphorylation *via* increased adenosine monophosphate (AMP)/adenosine triphosphate (ATP) ratio (30). Moreover, intragastric injection of acetate in mice showed a rapid increase in muscle AMP/ATP ratio and AMPK phosphorylation (31).

In addition to effects on skeletal muscle metabolism, acetate mediated upregulation of genes involved in fat oxidation (uncoupling protein 2, peroxisome proliferator-activated receptor alpha, and carnitine palmitoyl transferase 1) and increased *AMPK* phosphorylation in murine liver (32–34).

Although no actual substrate oxidation was measured, these studies suggest that acetate may increase liver and skeletal muscle oxidative capacity in an AMPK-mediated fashion, at least in rodent models. Our recently published human intervention study in overweight/obese normoglycemic men showed that acute distal, not proximal, colonic acetate infusions (180 mmol/L) increased fasting fat oxidation by around 25%, which may be possibly mediated *via* increased circulating acetate levels (7). Together, these data suggest that the observed *in vivo* effects on whole-body fat oxidation in overweight/obese humans may be partly mediated *via* a direct effect of circulating acetate on skeletal muscle fat oxidation. Therefore, we hypothesized that SA (sodium acetate) increases fat oxidation in a time- and dose-dependent manner in differentiated human primary muscle cells (HSkMC).

Fat oxidation was investigated by measuring SA effects on both endogenous (intracellular) and exogenous (extracellular) ^14^C-palmitate oxidation. Additionally, from a mechanistic perspective, we investigated SA-mediated effects on AMPK phosphorylation in HSkMC.

## Materials and Methods

HSkMC were isolated from rectus abdominal muscle tissue following surgery as described previously from a healthy male donor, insulin sensitive (HOMA-IR = 0.37), 45 year old with a BMI of 23.5 kg/m^2^ (**Figure S4**, **Table S1**) (35). Cells were proliferated using Dulbecco’s modified eagle medium (DMEM) low glucose (5.5 mmol/L) (#D6046-500 ml, Sigma-Aldrich) supplemented with 16% fetal bovine serum (FBS) (Batch BDC-11933, Bodinco BV), 0.05% bovine serum albumin (BSA) (#A4503-100 g, Sigma-Aldrich), 1 µmol/L dexamethasone (#D4902-100 mg, Sigma-Aldrich), 0.5 mg/ml bovine fetuin (#10505053, Thermo Fisher Scientific), 1× antibiotic/antimycotic (#15240-062, Thermo Fisher Scientific), and 0.01 µg/ml recombinant human epidermal growth factor (#PHG0311, Thermo Fisher Scientific). Cells were cultured in differentiation medium containing MEM-Alpha medium/Glutamax (#32561-029, Thermo Fisher Scientific) supplemented with 2% FBS, 0.5 mg/ml bovine fetuin, and 1× antibiotic/antimycotic. Antibodies against total AMPK*α*, and phosphorylated *Thr^172^-AMPKα* were purchased from Cell Signaling (#2603 and #2535, respectively MA, USA). Secondary antibodies (Goat-anti-Rabbit HRP) were purchased from Vector Labs (#1000). SA was purchased from Sigma-Aldrich (#S2889). For fat oxidation assay, we used **4-(2-**Hydroxyethyl) piperazine-1-ethanesulfonic acid (HEPES, #H3375, Sigma-Aldrich), L-carnitine hydrochloric acid (carnitine, #C0283, Sigma-Aldrich), ^14^C-palmitate (#NEC075H250uC, Perkin Elmer), non-labeled palmitate (#P0500, Sigma-Aldrich), and perchloric acid (#244252, Sigma-Aldrich).

### Cell Culture

HSkMC were cultivated in proliferation medium under 5% CO_2_ at 37°C. For differentiation towards functional myotubes, medium was changed to differentiation medium when reaching 80% confluence, as described above. At day 8–11 of differentiation viable myotubes were used for fat oxidation experiments.

### Cytotoxicity Assay

A fluorometric lactate dehydrogenase (LDH) assay was used for estimating the number of nonviable cells, as marker of cytotoxicity (#G7890, Promega). Differentiated cells were cultured without (0 mmol/L), or with physiological (0.1 mmol/L) and supraphysiological (1 and 5 mmol/L) concentrations of SA during 4 and 24 h (**Figure S1**). Fluorescence was measured using a Spectramax plate reader (Molecular Devices, CA, USA). Percentage of cytotoxicity was calculated in comparison to Triton X-100 (100% lysis positive control) with the same incubation times.

### Fat Oxidation

For measurement of *in vitro* fat oxidation cells were incubated with ^14^C- labeled palmitate (250 µCi/ml; PerkinElmer, Boston, MA, USA) and non-labeled palmitate. Palmitate was coupled to fatty acid free BSA (7.5%) together with HEPES (100 mmol/L) and carnitine (40 mmol/L).The label solution contained ^14^C-labeled palmitate (1 μCi/ml), 100 μM non-labeled (cold) palmitate, 0.25% BSA, 12.5 mM HEPES, and 1 mM L-Carnitine. After SA incubation, complete (^14^CO_2_) and incomplete (^14^ASM, acid soluble metabolites) oxidation products were measured, as previously described (36).

^14^ASM include acetyl-CoA, acetyl carnitines, ketone bodies, and TCA cycle intermediaries. Briefly, following incubation, medium was transferred to a custom-made Teflon 24-well CO_2_ trapping plate that was sealed, 70% perchloric acid was injected (Hamilton syringe, 1705N) through a silicon layer in the lid directly into the media. This moved the CO_2_ through a tunnel to an adjacent well where it was trapped in 1 N NaOH. After overnight trapping, complete and incomplete oxidation products were measured by Scintillation counting (using a Tri-Carb 2910 TR liquid scintillation analyzer, Perkin Elmer). For measuring endogenous fat oxidation, cells were pre-incubated for 24 h with ^14^C- labeled palmitate (250 µCi/ml; PerkinElmer, Boston, MA, USA) without carnitine and washed prior to SA incubations.

### Western Blotting

Following incubations without (0 mmol/L) or with SA (0.1 or 0.5 mmol/L), cells were washed with ice-cold PBS and lysed with RIPA buffer supplemented with protease (#04693132001, Sigma-Aldrich) and phosphatase (#04906845001, Sigma-Aldrich) inhibitors. Samples were vortexed, left on ice (~10 min) and snap-frozen in liquid nitrogen. Supernatants were subjected to western blot analysis. Briefly, a total of 10 µg of protein was loaded on a 4–12% SDS-PAGE gel using a Criterion XT precast gel (10–300 kilodaltons, Bio-Rad) with MOPS buffer (#161-0788, Biorad). After separation, proteins were transferred using a Criterion blot system (Bio-Rad) in 1xTG buffer (20% methanol + 0.05% SDS) onto a 0.45 µm nitrocellulose membrane (GE Healthcare, Netherlands), and membranes were blocked using tris-buffered saline (TBS)/3%BSA/0.1%Tween-20 [for phosphorylated antibodies and for non-phosphorylated antibodies they were blocked in TBS/5%non-fat dry milk (NFDM)/0.1%Tween-20]. Next, the membrane was incubated with primary antibodies (rabbit-anti human p-AMPK diluted 1:1,000 in TBS/3%BSA/0.1%Tween and rabbit-anti human AMPK in TBS/5%/NFDM/0.1%Tween-20) (Cell Signaling #2535 and #2603, respectively). After overnight incubations at 4°C, membranes were washed three times with tris-buffered saline tween (TBST) and incubated with HRP-conjugated secondary antibodies for 1 h (goat-anti-rabbit HRP) diluted 1:10,000 in TBS 3%BSA/0.1%Tween-20 or in TBS/5%NFDM/0.1%Tween-20.

Finally, after washing with TBST, last time with TBS, bands were visualized using ECL substrate (Super Signal West Femto, #34095, Thermo Scientific) according to the protocol supplied by the manufacturer using the Bio-Rad ChemiDoc imaging system (Biorad).

### Statistical Analysis

Values are expressed as mean and standard deviation. Significance was determined using the nonparametric Mann Whitney U-test when comparing two groups or the Kruskal-Wallis H-test, for multiple comparisons. In case of significant Kruskal-Wallis, Dunn’s *post hoc* test was performed.

Statistics were performed using the GraphPad Prism 5.0a software package (GraphPad Software, San Diego, CA, USA) and a P < 0.05 (two-sided P-value) was considered statistically significant.

## Results

### Time-Dependent Effect of SA on Exogenous and Endogenous Palmitate Oxidation

**Figure 1:**
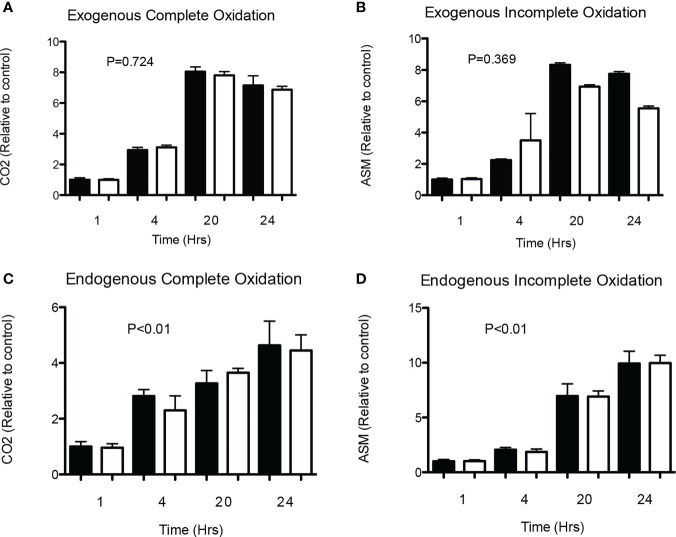
Time-effect of SA on exogenous and endogenous fat oxidation in HSkMC. **(A)** Complete (^14^CO_2_) and **(B)** incomplete (ASM) ^14^C-palmitate exogenous oxidation (2 independent experiments) was measured following 1, 4, 20 and 24 hours of incubation without (0 mmol/l) or with (0.1 mmol/l) SA. In addition, following 24h pre-incubation with ^14^C-palmitate, **(C)** complete (^14^CO_2_) and **(D)** incomplete (ASM) ^14^C-palmitate endogenous oxidation (2 independent experiments) was measured following 1, 4, 20- and 24-hours incubation without (0 mmol/l) or with (0.1 mmol/l) SA. Data expressed as mean and standard deviation and expressed as relative to control (black). P value corresponds to Kruskal-Wallis test. Post-hoc test showed no differences between control and acetate treated cells at each timepoint.

First of all, both acute (4 h) as well as chronic (24 h) incubation with physiological (0.1 mmol/L) and supraphysiological concentrations (1 and 5 mmol/L) of SA did not affect LDH activity, indicating no cytotoxic effect of SA in HSkMC (see **Figure S1**). Secondly, we investigated whether SA has a time-dependent effect on complete or incomplete exogenous/endogenous ^14^C-palmitate oxidation in differentiated HSkMC. Briefly, cells were incubated with a physiological concentration of SA (0.1 mmol/L) or without (0 mmol/L) for up to 24 h. As expected, and shown in **Figure 1**, an increase in fat oxidation was observed over time. However, both complete (**Figure 1A**) and incomplete (**Figure 1B**) exogenous fat oxidation were not affected when comparing SA to control treated cells after acute (1–4 h) and chronic (20–24 h) exposure. Next, to investigate the effect of SA on endogenous fat oxidation cells were first pre-incubated with ^14^C-palmitate for 24 h and after removal of the label, cells were incubated with SA (0.1 mmol/L) or without (0 mmol/L) for up to 24 h. Although we found a significant increase over time, no differences were observed between SA and control treated cells in complete (**Figure 1C**) and incomplete (**Figure 1D**) endogenous fat oxidation at each timepoint.

### Dose-Dependent Effect of SA on Exogenous or Endogenous Fat Oxidation

**Figure 2:**
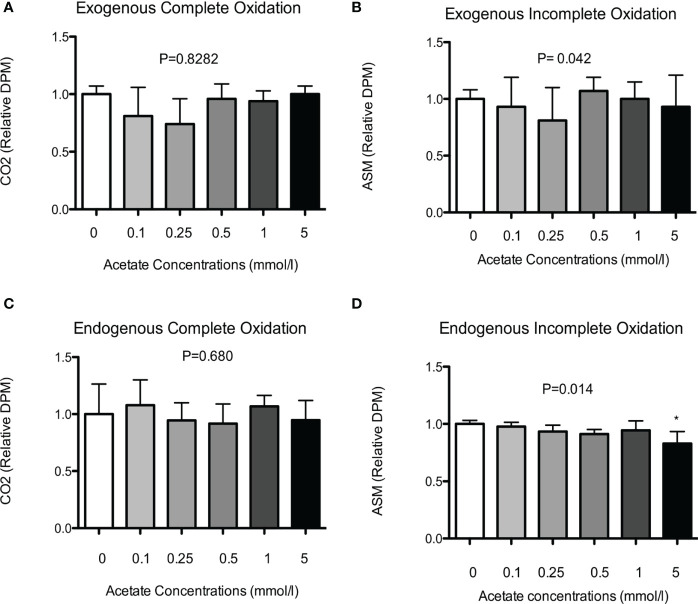
Dose-effect of SA on exogenous and endogenous fat oxidation in HSkMC. **(A)** Complete (^14^CO_2_) and **(B)** incomplete (ASM) ^14^C-palmitate exogenous oxidation (5 independent experiments) was measured following 20h incubation without (0 mmol/l) or with (0.1-5 mmol/l) SA. In addition, following 24h pre-incubation with ^14^C-palmitate, **(C)** complete (^14^CO_2_) and **(D)** incomplete (ASM) ^14^C-palmitate endogenous oxidation (4 independent experiments) was measured following 20h incubation without (0 mmol/l) or with (0.1-5 mmol/l) SA. Data expressed as mean and standard deviation and expressed as relative to control treated cells. P value corresponds to Kruskal-Wallis test. Post-hoc test significance is compared to control (0 mmol/l) indicated as asterisk (*) when P < 0.05.

Subsequently, we investigated the dose-effect of SA on exogenous fat oxidation in differentiated HSkMC. Cells were incubated without (0 mmol/L), or with physiological (0.1, 0.25, 0.5 mmol/L) and supraphysiological (1 and 5 mmol/L) concentrations of SA for up to 20 h. However, as shown in **Figure 2**, SA did neither increase complete (**Figure 2A**) nor incomplete (**Figure 2B**) exogenous fat oxidation as compared to control treated cells.

To investigate dose-dependent effect of SA on endogenous fat oxidation cells were first pre-incubated with ^14^C-palmitate for 24 h and after removal of the label, cells were treated without (0 mmol/L), or with physiological (0.1, 0.25, 0.5 mmol/L) and supraphysiological (1 and 5 mmol/L) concentrations of SA for up to 20 h. In line with the exogenous oxidation data, we did not observe a dose-dependent increase in complete (**Figure 2C**) palmitate oxidation as compared to control treated cells. However, we found a significant decrease in the incomplete oxidation (**Figure 2D**), but only following incubation with the highest dose (5 mmol/L) of SA (P < 0.05).

### Effect of SA on AMPK Phosphorylation

**Figure 3:**
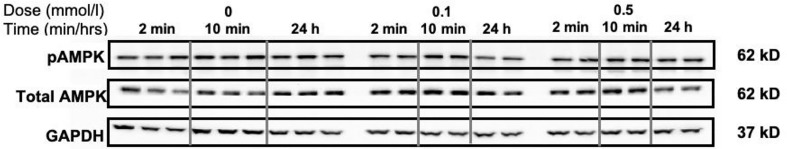
Time and dose-dependent effects of SA on total and pAMPK in *HSkMC*. Cells were incubated without (0 mmol/l) and with (0.1 and 0.5 mmol/l) SA for 2 or 10 min and up to 24 h. After incubation, cells were lysed with RIPA buffer supplemented with protease and phosphatase inhibitors and samples were subjected to western analysis for total *AMPKα (62kD band)*, phosphorylated AMPK (*Thr^172^-AMPKα*) and GAPDH (37kD band) was used as a loading control.

In line with our oxidation data, we did not observe a time (2 min, 10 min, and 24 h) or dose (0.1 and 0.5 mmol/L) dependent effect on total or phosphorylated *Thr^172^-AMPKα* content in HSkMC (**Figure 3**). Of interest, *Thr^172^-AMPKα* phosphorylation was increased in our HSkMC model following 4- and 6-h incubations with the major AMPK activator AICAR (1 mmol/L) (**Figure S3**). Nevertheless, this AICAR-mediated increase in AMPK phosphorylation was not accompanied by an increased complete nor incomplete exogenous/endogenous fat oxidation using AICAR (1 mmol/L) (**Figure S2**). In contrast, treatment of HSkMC using an inhibitor (etomoxir, 100 µmol/L) showed marked changes in complete and incomplete palmitate oxidation, indicating that our HSkMC is dynamic and fully functional (**Figure S2**).

## Discussion

Recently we showed that distal (not proximal) colonic infusions of acetate and SCFA mixtures (rich in acetate) increased circulating acetate levels (120 min) in overweight/obese humans (7, 37). Of note, we found a positive relationship between increments in acetate levels and fasting whole-body fat oxidation. Therefore, we hypothesized that circulating acetate may directly affect fat oxidation in human skeletal muscle contributing to the observed increments in *in vivo* whole-body fat oxidation. To our knowledge, this is the first study that investigates the direct (dose/time) effects of SA on fat oxidation (endo-exogenous) and whether effects are dependent on AMPK phosphorylation in a HSkMC model. However, our data showed no time- or dose-dependent increase of SA on either endo- or exogenous palmitate oxidation in HSkMC. In line, AMPK phosphorylation was not affected by acute nor chronic SA treatment.

*In vivo* colonic infusions (37, 38) showed an acute effect on fasting whole-body fat oxidation in overweight-obese men with acetate concentrations reaching average plasma acetate levels of 40 µmol/L. However, our *in vitro* experiments showed no dose- or time effect of SA, with physiological concentrations (0–0.5 mmol/L), reached after *in vivo* dietary fiber fermentation (10–13), and no effect with supraphysiological concentrations (1–5 mmol/L). Although the actual interstitial/intracellular concentration of acetate in the skeletal muscle remains unknown, it has been suggested in rats (39) and humans (40) that acetate uptake in skeletal muscle is proportional to circulating levels. In support, using labeled ^13^C SA in healthy lean individuals has shown ^13^CO_2_ breath recovery rates between 40–80%, which may indicate acetate uptake and utilization/oxidation in the TCA cycle of metabolically relevant organs (i.e. liver, muscle) (41–43). Of note, in our *in vitro* experiments SA was provided as a single bolus, and acetate may have been rapidly absorbed, and used into the tricarboxylic acid cycle (TCA) and saturate connecting metabolic pathways (44). In support, acute intravenous infusions of acetate (2.5 mmol per min for 1 h) in humans did not increase energy expenditure, which was partly explained by the fact that acetate might replace long chain fatty acids as preferred oxidation fuel in TCA-connected metabolic pathways (45). Of interest, a slight time-dependent decrease in endogenous (incomplete) oxidation was observed (**Figure 2D**), which reached significance following incubation with the highest dose of SA (5 mmol/L) compared to control cells, possibly indicating preferential use of SA as oxidation fuel. Future studies using labeled SA are needed in order to investigate the metabolic fate of acetate in human skeletal muscle. Moreover, whether metabolic effects of acetate in human skeletal muscle are GPR-dependent remains unclear given the inconclusive expression of GPR 41 in our HSkMC model (**Figure S5**) and therefore warrant further investigation of other GPRs (e.g. GPR43).

As mentioned above, our design (one single bolus of SA), may not mimic a continuous delivery of gut-derived acetate to the skeletal muscle. Acetate colonic absorption seems to be concentration dependent (46) and to gradually increase plasma levels, thus reaching skeletal muscle in a more continuous manner *in vivo*. Nevertheless, our doses were based on human prebiotic interventions that report circulating acetate concentrations up to 450 µmol/L (9–14). In support of a continuous supply, a 4-week supplementation of a high amount of fermentable (type 2) resistant starch (30 g/d *vs* rapidly digestible starch 20 g) increased acetate uptake in skeletal muscle (and adipose tissue) in healthy subjects (47). Although no fat oxidation was measured, the intervention improved peripheral insulin sensitivity (euglycemic hyperinsulinemic clamp) (47), thus potentially indicating that a continuous supply of gut-derived acetate into the muscle (instead of a single bolus) is needed to induce major metabolic effects.

Based on previous animal data, acetate (oral injection) acutely increases phosphorylation of *Thr^172^-AMPKα, at least* in rat skeletal muscle (*in vivo*) and *in vitro* using L6 myotubes (30, 31). The putative mechanism of acetate in the regulation of skeletal muscle fat oxidation, is through rapid (acute) catabolic conversion of acetate to acetyl CoA (mediated by acetyl Co A synthase), increasing the AMP/ATP ratio which subsequently increases AMPK phosphorylation. We replicated the exact same incubation time/dose as in above mentioned animal studies; however, we could not corroborate previous results. Importantly, our model is fully functional as observed by pronounced effects of Etomoxir.

Nevertheless, our model may not be the ideal system to investigate (acute) AMPK-dependent effects on fat oxidation (See **Figure S2**) and/or effects may be donor-specific (48, 49). Of note, AICAR administration in humans did not increase AMPK phosphorylation in skeletal muscle but increased hepatic fatty acid oxidation and lowered hepatic glucose production (50). Furthermore, AMPK-dependent fat oxidation might be limited in HSkMC and/or an AMPK-dependent effect on glucose uptake/oxidation may be preferentially activated (51, 52) as well as muscle fiber type-dependent activation of AMPK (53) in HSkMC. Although, acetate has shown AMPK-dependent fat oxidation in rodents, acetate-mediated effect on AMPK activation in skeletal muscle may be species-specific (54).

Lastly, circulating acetate may modulate other metabolically active tissues in humans that may explain the *in vivo* increments in whole-body fat oxidation. First, SA may increase liver/adipose tissue fat oxidative capacity, since *in vitro* and rodent studies have shown increments in phosphorylated AMPK and total AMPK (31, 32). In addition, acetate turnover rate is the highest in the liver of mammals as compared to other tissues (i.e. muscle and adipose tissues) indicating an important regulatory role of the liver in acetate metabolism (55). Second, other gut-derived metabolites such as butyrate may increase mitochondrial function and expression of fatty acid oxidative genes as observed in mice skeletal muscle (56, 57). Kinetic studies demonstrated that around 24% of colonic acetate is converted *via* bacterial cross-feeding into butyrate in metabolically healthy adults (58).

Finally, SCFA might affect the secretion of gut-derived hormones such as PYY and GLP-1, which have been associated with increased whole-body fat oxidation and energy expenditure in humans (59, 60). In line, data from our group and others reported increments of circulating PYY levels following colonic SCFA infusion in humans (37, 61). In agreement, a recent study reported that human skeletal muscle and muscle progenitor cells express PYY and its receptors, suggesting that our observed increase in whole-body fat oxidation might be partly explained *via* PYY-mediated effects on muscle fat oxidation (62). Our model showed no expression of the PYY receptor NPY2R during proliferation and differentiation (data not shown).

In addition, other gut-derived peptides warrant further investigation with respect to their respective roles on skeletal muscle fat oxidation under different metabolic status. For instance, ghrelin and gastric inhibitory polypeptide (GIP) may affect fat oxidation and thereby a protective role in lipid-induced skeletal muscle insulin resistance (63, 64). Moreover, fibroblast growth factor (FGF) 15/19 secreted from small intestine should be further investigated regarding their involvement in human skeletal muscle fat oxidation (65).

In conclusion, our data showed no time- or dose-dependent increase of SA on endogenous or exogenous palmitate oxidation as well as no increments in AMPK phosphorylation following acute/chronic SA treatment in our human primary muscle cell model HSkMC derived from a lean insulin sensitive male donor. However, we cannot exclude that our previously reported *in vivo* effect of colonic acetate administration on fat oxidation in overweight individuals might be partly explained by the effect of other gut-derived metabolites and their signaling pathways (i.e. *via* PYY) on muscle fat oxidation or by direct effects of SCFA (i.e. butyrate) on other tissues (i.e. liver and adipose tissue). Furthermore, fat oxidation might be donor-dependent and/or species-specific.

## Data Availability Statement

The raw data supporting the conclusions of this article will be made available by the authors, without undue reservation.

## Ethics Statement

The studies involving human participants were reviewed and approved by Medical Ethical Committee Jessa Hospital, Hasselt, and Hasselt University, Belgium. The patients/participants provided their written informed consent to participate in this study.

## Author Contributions

MG, JJ, EC, and EB were responsible for the study concept and design, analysis and interpretation of the data, and critical revision of the manuscript for important intellectual content. MG, YE, and NH generated data. MG acquired all data, completed statistical analysis, and drafted the manuscript. EB obtained funding and supervised the study. All authors contributed to the article and approved the submitted version.

## Funding

The study was supported by a Kootstra Talent grant from Maastricht University Medical Centre^+^ (Applicant: EB, Candidate: EC) and MG’s salary was paid by a grant from Consejo Nacional de Ciencia y Tecnología (CONACYT). The funders had no role in study design, data collection and analysis, decision to publish, or preparation of the manuscript.

## Conflict of Interest

The authors declare that the research was conducted in the absence of any commercial or financial relationships that could be construed as a potential conflict of interest.

## References

[1] Engin A. The Definition and Prevalence of Obesity and Metabolic Syndrome. Adv Exp Med Biol (2017) 960:1–17. 10.1007/978-3-319-48382-5_1 28585193

[2] Stinkens R Goossens GH Jocken JW Blaak EE. Targeting Fatty Acid Metabolism to Improve Glucose Metabolism. Obes Rev Off J Int Assoc Study Obes (2015) 16(9):715–57. 10.1111/obr.12298 26179344

[3] Canfora EE Jocken JW Blaak EE. Short-Chain Fatty Acids in Control of Body Weight and Insulin Sensitivity. Nat Rev Endocrinol (2015) 11(10):577–91. 10.1038/nrendo.2015.128 26260141

[4] Canfora EE Meex RCR Venema K Blaak EE. Gut Microbial Metabolites in Obesity, NAFLD and T2DM. Nat Rev Endocrinol (2019) 15(5):261–73. 10.1038/s41574-019-0156-z 30670819

[5] Conlon MA Bird AR. The Impact of Diet and Lifestyle on Gut Microbiota and Human Health. Nutrients (2015) 7(1):17–44. 10.3390/nu7010017 PMC4303825 25545101

[6] Marchesi JR Adams DH Fava F Hermes GDA Hirschfield GM Hold G. The Gut Microbiota and Host Health: A New Clinical Frontier. Gut (2015) 65(2):330–9. 10.1136/gutjnl-2015-309990 PMC4752653 26338727

[7] Canfora EE Blaak EE. Acetate: A Diet-Derived Key Metabolite in Energy Metabolism: Good or Bad in Context of Obesity and Glucose Homeostasis? Curr Opin Clin Nutr Metab Care (2017) 20(6):477–3. 10.1097/MCO.0000000000000408 28795972

[8] Cummings JH Pomare EW Branch WJ Naylor CP Macfarlane GT. Short Chain Fatty Acids in Human Large Intestine, Portal, Hepatic and Venous Blood. Gut (1987) 28(10):1221–7. 10.1136/gut.28.10.1221 PMC1433442 3678950

[9] Müller M Hernández MAG Goossens GH Reijnders D Holst JJ Jocken JWE. Circulating But Not Faecal Short-Chain Fatty Acids Are Related to Insulin Sensitivity, Lipolysis and GLP-1 Concentrations in Humans. Sci Rep (2019) 9(1):12515. 10.1038/s41598-019-48775-0 31467327 PMC6715624

[10] Ferchaud-Roucher V Pouteau E Piloquet H Zair Y Krempf M. Colonic Fermentation From Lactulose Inhibits Lipolysis in Overweight Subjects. Am J Physiol Endocrinol Metab (2005) 289(4):E716–20. 10.1152/ajpendo.00430.2004 16150956

[11] Pouteau E Vahedi K Messing B Flourie B Nguyen P Darmaun D. Production Rate of Acetate During Colonic Fermentation of Lactulose: A Stable-Isotope Study in Humans. Am J Clin Nutr (1998) 68(6):1276–83. 10.1093/ajcn/68.6.1276 9846859

[12] Luo J Rizkalla SW Alamowitch C Boussairi A Blayo A Barry JL. Chronic Consumption of Short-Chain Fructooligosaccharides by Healthy Subjects Decreased Basal Hepatic Glucose Production But Had No Effect on Insulin-Stimulated Glucose Metabolism. Am J Clin Nutr (1996) 63(6):939–45. 10.1093/ajcn/63.6.939 8644690

[13] Boll EV Ekstrom LM Courtin CM Delcour JA Nilsson AC Bjorck IM. Effects of Wheat Bran Extract Rich in Arabinoxylan Oligosaccharides and Resistant Starch on Overnight Glucose Tolerance and Markers of Gut Fermentation in Healthy Young Adults. Eur J Nutr (2016) 55(4):1661–70. 10.1007/s00394-015-0985-z 26169871

[14] Canfora EE van der Beek CM Hermes GDA Goossens GH Jocken JWE Holst JJ. Supplementation of Diet With Galacto-Oligosaccharides Increases Bifidobacteria, But Not Insulin Sensitivity, in Obese Prediabetic Individuals. Gastroenterology (2017) 153(1):87–97.e3. 10.1053/j.gastro.2017.03.051 28396144

[15] Neis EPJG van Eijk HMH Lenaerts K Olde Damink SWM Blaak EE Dejong CHC. Distal Versus Proximal Intestinal Short-Chain Fatty Acid Release in Man. Gut (2019) 68(4):764. 10.1136/gutjnl-2018-316161 29618497

[16] Le Poul E Loison C Struyf S Springael JY Lannoy V Decobecq ME. Functional Characterization of Human Receptors for Short Chain Fatty Acids and Their Role in Polymorphonuclear Cell Activation. J Biol Chem (2003) 278(28):25481–9. 10.1074/jbc.M301403200 12711604

[17] Brown AJ Goldsworthy SM Barnes AA Eilert MM Tcheang L Daniels D. The Orphan G Protein-Coupled Receptors GPR41 and GPR43 Are Activated by Propionate and Other Short Chain Carboxylic Acids. J Biol Chem (2003) 278(13):11312–9. 10.1074/jbc.M211609200 12496283

[18] Tang C Ahmed K Gille A Lu S Grone H-J Tunaru S. Loss of FFA2 and FFA3 Increases Insulin Secretion and Improves Glucose Tolerance in Type 2 Diabetes. Nat Med (2015) 21(2):173–7. 10.1038/nm.3779 25581519

[19] Priyadarshini M Villa SR Fuller M Wicksteed B Mackay CR Alquier T. Ffar2, Regulates Insulin Secretion. Mol Endocrinol (2015) 29(7):1055–66. 10.1210/me.2015-1007 PMC4484778 26075576

[20] Ge H Li X Weiszmann J Wang P Baribault H Chen J-L. Activation of G Protein-Coupled Receptor 43 in Adipocytes Leads to Inhibition of Lipolysis and Suppression of Plasma Free Fatty Acids. Endocrinology (2008) 149(9):4519–26. 10.1210/en.2008-0059 18499755

[21] Aberdein N Schweizer M Ball D. Sodium Acetate Decreases Phosphorylation of Hormone Sensitive Lipase in Isoproterenol-Stimulated 3T3-L1 Mature Adipocytes. Adipocyte (2014) 3(2):121–5. 10.4161/adip.27936 PMC3979876 24719785

[22] Jocken JWE González Hernández MA Hoebers NTH van der Beek CM Essers YPG Blaak EE. Short-Chain Fatty Acids Differentially Affect Intracellular Lipolysis in a Human White Adipocyte Model. Front Endocrinol (2018) 8(372). 10.3389/fendo.2017.00372 PMC5768634 29375478

[23] Sahuri-Arisoylu M Brody LP Parkinson JR Parkes H Navaratnam N Miller AD. Reprogramming of Hepatic Fat Accumulation and ‘Browning’ of Adipose Tissue by the Short-Chain Fatty Acid Acetate. Int J Obes (2005) (2016) 40(6):955–63. 10.1038/ijo.2016.23 26975441

[24] Crouse JR Gerson CD DeCarli LM Lieber CS. Role of Acetate in the Reduction of Plasma Free Fatty Acids Produced by Ethanol in Man. J Lipid Res (1968) 9(4):509–12. 10.1016/S0022-2275(20)42731-2 5725882

[25] Laurent C Simoneau C Marks L Braschi S Champ M Charbonnel B. Effect of Acetate and Propionate on Fasting Hepatic Glucose Production in Humans. Eur J Clin Nutr (1995) 49(7):484–91. 7588498

[26] Wolever TM Brighenti F Royall D Jenkins AL Jenkins DJ. Effect of Rectal Infusion of Short Chain Fatty Acids in Human Subjects. Am J Gastroenterol (1989) 84(9):1027–33. 2773895

[27] Suokas A Kupari M Heikkila J Lindros K Ylikahri R. Acute Cardiovascular and Metabolic Effects of Acetate in Men. Alcohol Clin Exp Res (1988) 12(1):52–8. 10.1111/j.1530-0277.1988.tb00132.x 3279860

[28] Keenan MJ Zhou J McCutcheon KL Raggio AM Bateman HG Todd E. Effects of Resistant Starch, a Non-Digestible Fermentable Fiber, on Reducing Body Fat. Obes (Silver Spring Md) (2006) 14(9):1523–34. 10.1038/oby.2006.176 17030963

[29] Cani PD Dewever C Delzenne NM. Inulin-Type Fructans Modulate Gastrointestinal Peptides Involved in Appetite Regulation (Glucagon-Like Peptide-1 and Ghrelin) in Rats. Br J Nutr (2004) 92(3):521–6. 10.1079/BJN20041225 15469657

[30] Maruta H Yoshimura Y Araki A Kimoto M Takahashi Y Yamashita H. Activation of AMP-Activated Protein Kinase and Stimulation of Energy Metabolism by Acetic Acid in L6 Myotube Cells. PloS One (2016) 11(6):e0158055. 10.1371/journal.pone.0158055 PMC4922563 27348124

[31] Yamashita H Maruta H Jozuka M Kimura R Iwabuchi H Yamato M. Effects of Acetate on Lipid Metabolism in Muscles and Adipose Tissues of Type 2 Diabetic Otsuka Long-Evans Tokushima Fatty (OLETF) Rats. Biosci Biotechnol Biochem (2009) 73(3):570–6. 10.1271/bbb.80634 19270372

[32] Sakakibara S Yamauchi T Oshima Y Tsukamoto Y Kadowaki T. Acetic Acid Activates Hepatic AMPK and Reduces Hyperglycemia in Diabetic KK-A(y) Mice. Biochem Biophys Res Commun (2006) 344(2):597–604. 10.1016/j.bbrc.2006.03.176 16630552

[33] Kondo T Kishi M Fushimi T Kaga T. Acetic Acid Upregulates the Expression of Genes for Fatty Acid Oxidation Enzymes in Liver to Suppress Body Fat Accumulation. J Agric Food Chem (2009) 57(13):5982–6. 10.1021/jf900470c 19469536

[34] Smith PM Howitt MR Panikov N Michaud M Gallini CA Bohlooly YM. The Microbial Metabolites, Short-Chain Fatty Acids, Regulate Colonic Treg Cell Homeostasis. Sci (New York NY) (2013) 341(6145):569–73. 10.1126/science.1241165 PMC3807819 23828891

[35] Verboven K Wouters K Gaens K Hansen D Bijnen M Wetzels S. Abdominal Subcutaneous and Visceral Adipocyte Size, Lipolysis and Inflammation Relate to Insulin Resistance in Male Obese Humans. Sci Rep (2018) 8(1):4677. 10.1038/s41598-018-22962-x 29549282 PMC5856747

[36] Hulver MW Berggren JR Carper MJ Miyazaki M Ntambi JM Hoffman EP. Elevated Stearoyl-CoA Desaturase-1 Expression in Skeletal Muscle Contributes to Abnormal Fatty Acid Partitioning in Obese Humans. Cell Metab (2005) 2(4):251–61. 10.1016/j.cmet.2005.09.002 PMC4285571 16213227

[37] van der Beek CM Canfora EE Lenaerts K Troost FJ Damink S Holst JJ. Distal, Not Proximal, Colonic Acetate Infusions Promote Fat Oxidation and Improve Metabolic Markers in Overweight/Obese Men. Clin Sci (Lond) (2016) 130(22):2073–82. 10.1042/CS20160263 27439969

[38] Canfora EE van der Beek CM Jocken JWE Goossens GH Holst JJ Olde Damink SWM. Colonic Infusions of Short-Chain Fatty Acid Mixtures Promote Energy Metabolism in Overweight/Obese Men: A Randomized Crossover Trial. Sci Rep (2017) 7(1):2360. 10.1038/s41598-017-02546-x 28539646 PMC5443817

[39] Karlsson N Fellenius E Kiessling KH. The Metabolism of Acetate in the Perfused Hind-Quarter of the Rat. Acta Physiol Scand (1975) 93(3):391–400. 10.1111/j.1748-1716.1975.tb05828.x 1146580

[40] Skutches CL Holroyde CP Myers RN Paul P Reichard GA. Plasma Acetate Turnover and Oxidation. J Clin Invest (1979) 64(3):708–13. 10.1172/JCI109513 PMC372171 468985

[41] Mittendorfer B Sidossis LS Walser E Chinkes DL Wolfe RR. Regional Acetate Kinetics and Oxidation in Human Volunteers. Am J Physiol Endocrinol Metab (1998) 274(6):E978–83. 10.1152/ajpendo.1998.274.6.E978 9611145

[42] Pouteau E Piloquet H Maugeais P Champ M Dumon H Nguyen P. Kinetic Aspects of Acetate Metabolism in Healthy Humans Using [1-13C] Acetate. Am J Physiol Endocrinol Metab (1996) 271(1):E58–64. 10.1152/ajpendo.1996.271.1.E58 8760082

[43] Wolfe RR Jahoor F. Recovery of Labeled CO2 During the Infusion of C-1- vs C-2-Labeled Acetate: Implications for Tracer Studies of Substrate Oxidation. Am J Clin Nutr (1990) 51(2):248–52. 10.1093/ajcn/51.2.248 2106256

[44] Neavyn MJ Boyer EW Bird SB Babu KM. Sodium Acetate as a Replacement for Sodium Bicarbonate in Medical Toxicology: A Review. J Med Toxicol (2013) 9(3):250–4. 10.1007/s13181-013-0304-0 PMC3771004 23636658

[45] Akanji AO Bruce MA Frayn KN. Effect of Acetate Infusion on Energy Expenditure and Substrate Oxidation Rates in Non-Diabetic and Diabetic Subjects. Eur J Clin Nutr (1989) 43(2):107–15. 2651106

[46] Ruppin H Bar-Meir S Soergel KH Wood CM Schmitt MG Jr. Absorption of Short-Chain Fatty Acids by the Colon. Gastroenterology (1980) 78(6):1500–7. 10.1016/S0016-5085(19)30508-6 6768637

[47] Robertson MD Bickerton AS Dennis AL Vidal H Frayn KN. Insulin-Sensitizing Effects of Dietary Resistant Starch and Effects on Skeletal Muscle and Adipose Tissue Metabolism. Am J Clin Nutr (2005) 82(3):559–67. 10.1093/ajcn/82.3.559 16155268

[48] Bajpeyi S Myrland CK Covington JD Obanda D Cefalu WT Smith SR. Lipid in Skeletal Muscle Myotubes Is Associated to the Donors’ Insulin Sensitivity and Physical Activity Phenotypes. Obes (Silver Spring Md) (2014) 22(2):426–34. 10.1002/oby.20556 PMC3883809 23818429

[49] Ukropcova B McNeil M Sereda O de Jonge L Xie H Bray GA. Dynamic Changes in Fat Oxidation in Human Primary Myocytes Mirror Metabolic Characteristics of the Donor. J Clin Invest (2005) 115(7):1934–41. 10.1172/JCI24332 PMC1159139 16007256

[50] Boon H Bosselaar M Praet SF Blaak EE Saris WH Wagenmakers AJ. Intravenous AICAR Administration Reduces Hepatic Glucose Output and Inhibits Whole Body Lipolysis in Type 2 Diabetic Patients. Diabetologia (2008) 51(10):1893–900. 10.1007/s00125-008-1108-7 18709353

[51] Sakoda H Ogihara T Anai M Fujishiro M Ono H Onishi Y. Activation of AMPK Is Essential for AICAR-Induced Glucose Uptake by Skeletal Muscle But Not Adipocytes. Am J Physiol Endocrinol Metab (2002) 282(6):E1239–44. 10.1152/ajpendo.00455.2001 12006353

[52] Musi N Goodyear LJ. AMP-Activated Protein Kinase and Muscle Glucose Uptake. Acta Physiol Scand (2003) 178(4):337–45. 10.1046/j.1365-201X.2003.01168.x 12864738

[53] Tobias IS Lazauskas KK Siu J Costa PB Coburn JW Galpin AJ. Sex and Fiber Type Independently Influence AMPK, TBC1D1, and TBC1D4 at Rest and During Recovery From High-Intensity Exercise in Humans. J Appl Physiol (Bethesda Md 1985) (2020) 128(2):350–61. 10.1152/japplphysiol.00704.2019 31895596

[54] Kjøbsted R Hingst JR Fentz J Foretz M Sanz M-N Pehmøller C. AMPK in Skeletal Muscle Function and Metabolism. FASEB J Off Publ Fed Am Soc Exp Biol (2018) 32(4):1741–77. 10.1096/fj.201700442R PMC5945561 29242278

[55] Knowles SE Jarrett IG Filsell OH Ballard FJ. Production and Utilization of Acetate in Mammals. Biochem J (1974) 142(2):401–11. 10.1042/bj1420401 PMC1168292 4441381

[56] Hong J Jia Y Pan S Jia L Li H Han Z. Butyrate Alleviates High Fat Diet-Induced Obesity Through Activation of Adiponectin-Mediated Pathway and Stimulation of Mitochondrial Function in the Skeletal Muscle of Mice. Oncotarget (2016) 7(35):56071–82. 10.18632/oncotarget.11267 PMC5302897 27528227

[57] Frampton J Murphy K Frost G Chambers E. Short-Chain Fatty Acids as Potential Regulators of Skeletal Muscle Metabolism and Function. Nat Metab (2020) 2:840–8. 10.1038/s42255-020-0188-7 32694821

[58] Boets E Gomand SV Deroover L Preston T Vermeulen K De Preter V. Systemic Availability and Metabolism of Colonic-Derived Short-Chain Fatty Acids in Healthy Subjects: A Stable Isotope Study. J Physiol (2017) 595(2):541–55. 10.1113/JP272613 PMC5233652 27510655

[59] Pannacciulli N Bunt JC Koska J Bogardus C Krakoff J. Higher Fasting Plasma Concentrations of Glucagon-Like Peptide 1 Are Associated With Higher Resting Energy Expenditure and Fat Oxidation Rates in Humans. Am J Clin Nutr (2006) 84(3):556–60. 10.1093/ajcn/84.3.556 16960169

[60] Karra E Chandarana K Batterham RL. The Role of Peptide YY in Appetite Regulation and Obesity. J Physiol (2009) 587(1):19–25. 10.1113/jphysiol.2008.164269 19064614 PMC2670018

[61] Freeland KR Wolever TMS. Acute Effects of Intravenous and Rectal Acetate on Glucagon-Like Peptide-1, Peptide YY, Ghrelin, Adiponectin and Tumour Necrosis Factor-Alpha. Br J Nutr (2010) 103(3):460–6. 10.1017/S0007114509991863 19818198

[62] Gheller BJ Blum JE Merritt EK Cummings BP Thalacker-Mercer AE. Peptide YY (Pyy) Is Expressed in Human Skeletal Muscle Tissue and Expanding Human Muscle Progenitor Cells. Front Physiol (2019) 10:188. 10.3389/fphys.2019.00188 30890955 PMC6412030

[63] Cervone DT Hucik B Lovell AJ Dyck DJ. Unacylated Ghrelin Stimulates Fatty Acid Oxidation to Protect Skeletal Muscle Against Palmitate-Induced Impairment of Insulin Action in Lean But Not High-Fat Fed Rats. Metab Open (2020) 5:100026. 10.1016/j.metop.2020.100026 PMC7424793 32812929

[64] Zhou H Yamada Y Tsukiyama K Miyawaki K Hosokawa M Nagashima K. Gastric Inhibitory Polypeptide Modulates Adiposity and Fat Oxidation Under Diminished Insulin Action. Biochem Biophys Res Commun (2005) 335(3):937–42. 10.1016/j.bbrc.2005.07.164 16105663

[65] Guan D Zhao L Chen D Yu B Yu J. Regulation of Fibroblast Growth Factor 15/19 and 21 on Metabolism: In the Fed or Fasted State. J Trans Med (2016) 14(1):63. 10.1186/s12967-016-0821-0 PMC4774037 26931208

