# Supplementary material for: Acetate Does Not Affect Palmitate Oxidation and AMPK Phosphorylation in Human Primary Skeletal Muscle Cells
Source: Front Endocrinol (Lausanne). 2021 Jun 17;12:659928. doi: 10.3389/fendo.2021.659928 (PMC8248488; doi:10.3389/fendo.2021.659928)
Supplement: Supplementary file 1 [file DataSheet_1.docx]

**Supplementary material**

 **Supplementary figure 1. Cytotoxicity effect of SA on *HSkMC*.** Cytotoxicity percentage without (0 mmol/l) and with (0.1, 1 and 5 mmol/l) SA following 4- and 24-hour incubations. Data expressed as mean and standard deviation. Group differences were tested with Kruskal-Wallis. Statistical significance compared to control (0mmol/l) indicated as asterisk (*) when P<0.05. Percentage of cytotoxicity after SA was calculated in comparison to Triton-X 100, which is a positive control (100% lysis).

**Supplementary figure 2. Muscle cell model exogenous and endogenous fat oxidation fat oxidation in HSkMC. (A)** Complete (^14^CO_2_) and **(B)** incomplete (ASM) ^14^C-palmitate exogenous oxidation was measured following incubation with AICAR (1 mmol/l) and etomoxir (100 μmol/l) (1 experiment). Next, after 24h pre-incubation with ^14^C-palmitate, **(C)** complete (^14^CO_2_) and **(D)** incomplete (ASM) ^14^C-palmitate endogenous fat oxidation was measured following incubation with AICAR (1 mmol/l) and etomoxir (100 μmol/l). Data expressed as relative to control treated cells. Statistical significance using Mann-Whitney test compared to control (0 mmol/l) indicated as asterisk (*) when P<0.05


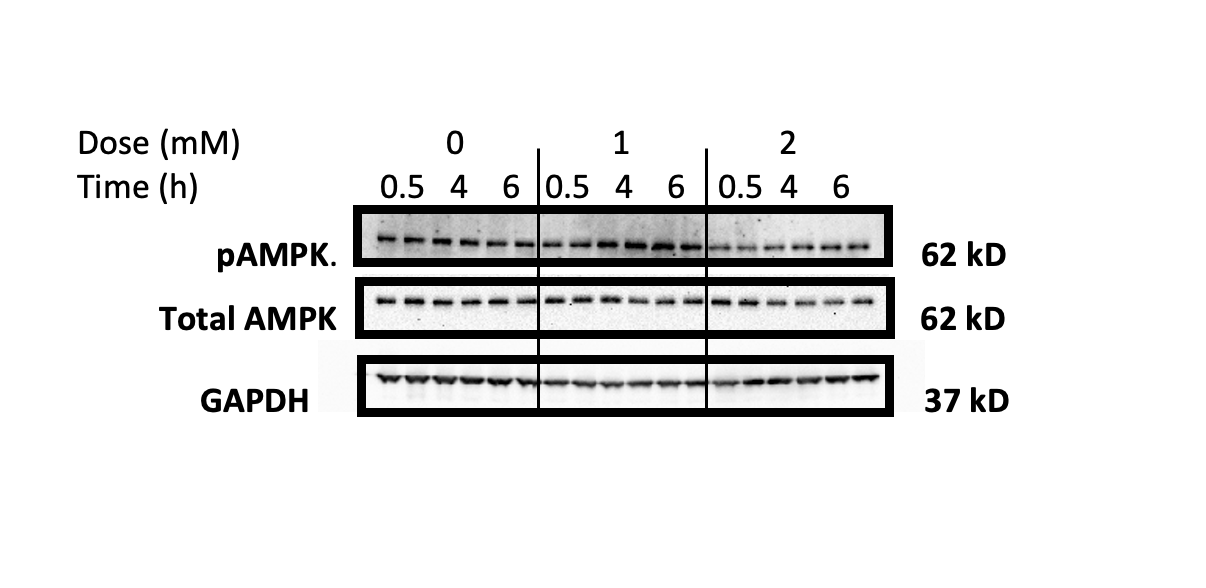


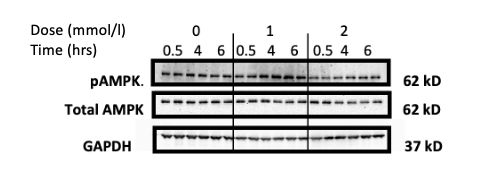


**Supplementary figure 3.** **Time dependent effect of AICAR on AMPK phosphorylation in HSkMC.** Control and AICAR (1 and 2 mmol/l) treated cells following 0.5, 4- and 6-hours incubations. After incubation, cells were lysed with RIPA buffer and samples were subjected to western analysis for phosphorylated AMPK *(*Thr^172^*-*AMPKα, 62kD band), total AMPKα (62 kD band) and GAPDH (*37kD* band) was used as a loading control.

| **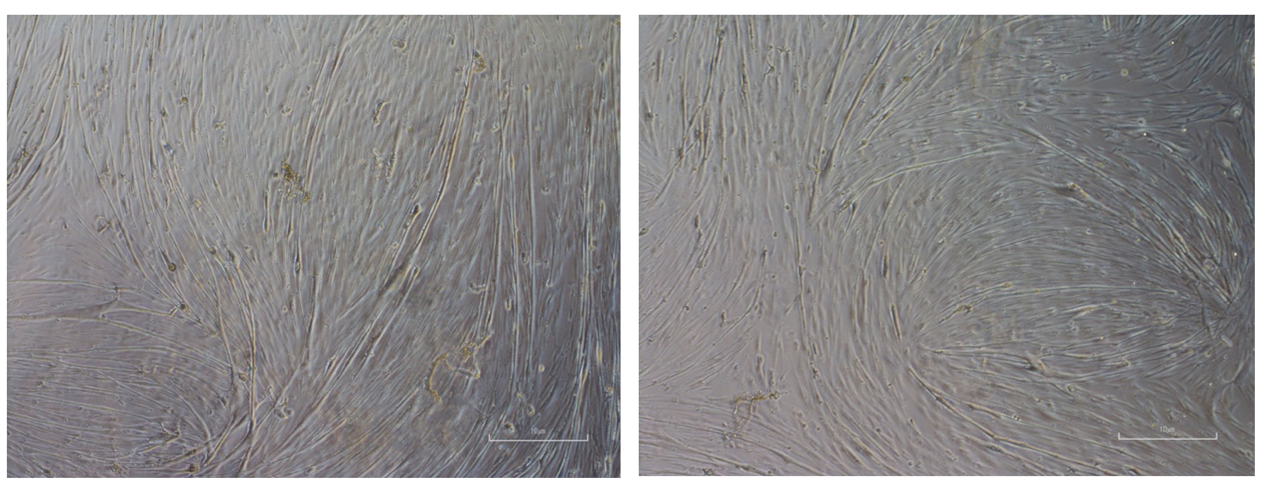** |  |
| --- | --- |
| **Supplementary figure 4. Fully differentiated myotubes at day 8-11 of differentiation**. Pictures taken immediately prior to fat oxidation experiments or cell lysis for western blot.  **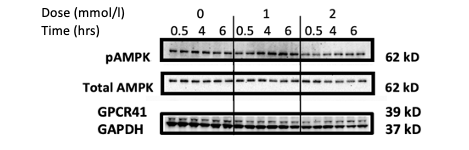**  **Supplementary figure 5.** **Time dependent effect of AICAR on AMPK phosphorylation in HSkMC.** Control and AICAR (1 and 2 mmol/l) treated cells following 0.5, 4- and 6-hours incubations. After incubation, cells were lysed with RIPA buffer and samples were subjected to western analysis for phosphorylated AMPK *(*Thr^172^*-*AMPKα, 62kD band), total AMPKα (62 kD band), GPCR41 (39kD) and GAPDH (*37kD* band) was used as a loading control. AICAR; 5-Aminoimidazole-4-carboxamide ribonucleotide, GPCR; g-protein coupled receptor, GAPDH; Glyceraldehyde 3-phosphate dehydrogenase, AMPK; AMP-activated protein kinase | |
|  | |

| **Supplementary Table. Donor characteristics** | |
| --- | --- |
| **Parameter** | **Value** |
| Age (yrs) | 45 |
| Length (m) | 1.9 |
| Weight (kg) | 85.0 |
| BMI (kg/m^2^) | 23.5 |
| Waist-Hip ratio | 0.95 |
| Systolic blood pressure (mmHg) | 120 |
| Diastolic blood pressure (mmHg) | 70 |
| Glucose (mmol/L) | 2.44 |
| Insulin (mU/L) | 3.50 |
| HOMA-IR | 0.380 |
| HbA1c % | 5.2 |
| Body Fat % | 19.1 |
| Fat mass (kg) | 16.2 |
| Fat free mass (kg) | 68.8 |
| Type of surgery | Inguinal hernia |
